# Implementation of Home Hospitalization and Early Discharge as an Integrated Care Service: A Ten Years Pragmatic Assessment

**DOI:** 10.5334/ijic.3431

**Published:** 2018-05-16

**Authors:** Carme Hernández, Jesus Aibar, Nuria Seijas, Imma Puig, Albert Alonso, Judith Garcia-Aymerich, Josep Roca

**Affiliations:** 1 Hospital Clínic de Barcelona, Institut d’Investigacions Biomèdiques August Pi i Sunyer, Centro de Investigación Biomédica en red, Enfermedades Respiratorias, University of Barcelona, Catalonia, ES; 2 Hospital Clínic de Barcelona, University of Barcelona, Barcelona, Catalonia, ES; 3 Hospital Clinic, Barcelona, Catalonia, ES; 4 Centre de Recerca en Epidemiologia Ambiental. Centro de Investigación Biomédica en red de Epidemiologia y Salud Pública, Universitat Pompeu Fabra, Barcelona, Catalonia, ES

**Keywords:** Home Hospitalization, chronic patients, integrated care, telemedicine, transitional care

## Abstract

Home Hospitalization has proven efficacy, but its effectiveness and potential as an Integrated Care Service in a real world setting deserves to be explored.

**Objective:**

To evaluate implementation and 10 years follow-up of Home Hospitalization and Early Discharge as an Integrated Care Service in an urban healthcare district in Barcelona.

**Methods:**

Prospective study with pragmatic assessment. Patients: Surgical and medical acute and exacerbated chronic patients requiring admission into a highly specialized hospital, from 2006 to 2015. Intervention: Home-based individualized care plan, administered as a hospital-based outreach service, aiming at substituting hospitalization and implementing a transitional care strategy for optimal discharge. Main measurements: Emergency Department, readmissions and mortality. Patients’ and professionals’ perspectives, technologies and costs were evaluated.

**Results:**

4,165 admissions (71 ± 15 yrs; Charlson Index 4 ± 3). In-hospital stay was 1 (0–3) days and the length of home-based stay was 6 (5–7) days. The 30-day readmission rate was 11% and mortality was 2%. Patients, careers and health professionals expressed high levels of satisfaction (98%). At the start, the service was reimbursed at a flat rate of 918€ per patient discharged, significantly lower than conventional hospitalization (2,879€) but still allowing the hospital to keep a balanced budget. At present, there is no difference in the payment schemes for both types of services.

**Conclusions:**

The service freed an average of 6 in-hospital days per patient. The program showed health value generation, as well as potential for synergies with community-based Integrated Care Services.

## Introduction

Since the early report by Leff et al [1] showing feasibility and cost-effectiveness of home hospitalization, assessment of Hospital at Home programs has historically shown significant complexities due to heterogeneities of the study groups, as well as to suboptimal definition of the interventions. It is of note, however, that recent reports [2,3,4,5,6] seem to overcome those limitations by demonstrating maturity and health value generation of Home Hospitalization/Early Discharge. Likewise, the 2016 Cochrane review [7] concludes that Home Hospitalization may constitute an effective alternative to inpatient care for a selected group of elderly patients requiring hospital admission.

In spite of successful experiences in specific settings [1,3,4,6], the role of Home Hospitalization and Early Discharge in an integrated care scenario requires further analysis. Moreover, reporting on real experiences of Home Hospitalization/Early Discharge deployed in locations served by heterogeneous healthcare providers, as done in the current report, is infrequent.

Catalonia (7.5 M inhabitants) [8] has a tax-based health care system that provides universal healthcare coverage. The Catalan Health Care System differs from other Spanish autonomous regions in the way it is organised: a unique network with a single public payer and multiple service providers publicly or privately owned. As it is the case in other Western European countries, the Catalan Health Care system is being strained by growing demands for healthcare and social care resulting from the increase in the numbers of old people. Since 2011, the successive Health Plans for Catalonia have included strategies to address these challenges by prioritising new models of healthcare delivery such as chronic patient care, home healthcare and home hospitalisation in addition to the empowerment of patients and carers. The experience collected in this paper was an early development of one of such models and is, overall, in alignment with the Health Plan strategies.

In 2006, the Hospital Clinic of Barcelona, a tertiary university hospital, initiated the deployment of integrated care services in one of the four healthcare sectors of the city (520.000 inhabitants), adopting the Chronic Care Model as the conceptual reference [9,10]. The program aimed at assessing the practicalities of the deployment of four integrated care services. These services were chosen because their adequate articulation can cover the longitudinal care requirements of the entire spectrum of severity of chronic patients. Home Hospitalization/Early Discharge was one of these services; being the remaining ones: i) Promotion of healthy life styles [11]; ii) Community-based prevention of hospitalizations [12]; and, iii) Transfer of specialized diagnosis to Primary Care [13,14], as described elsewhere [15,16]. A second aim was to assess transferability of the approach to two other European sites like Trondheim and Athens [16].

Home Hospitalization and Early Discharge programs have been mostly reported as stand-alone initiatives having poor or no interactions with community-based services. Instead, the current report addresses the service as part of transitional care strategies between hospital care and community-based services [12,17,18]. Transitional care strategies apply to at risk populations as they move from one level of care to another across different Healthcare providers. These strategies focus on time limited interventions that ensure continuity of care in these patients. Transitional care should always follow discharge from home-hospitalisation.

The current research was done in a real world setting with heterogeneous healthcare providers. Two explanatory studies done at Hospital Clinic before 2005 clearly demonstrated efficacy of both Home Hospitalization/Early Discharge [19] and transitional care strategies [17] in chronic respiratory patients. However, key factors limiting effectiveness of the interventions were demonstrated by further research [12]. Accordingly, the need for assessment of integrated care services in a real world setting was clearly identified.

We hypothesized that implementation of Home Hospitalization/Early Discharge for acute medical/surgical patients in a real world scenario shows high potential to generate healthcare efficiencies. To verify this hypothesis we adopted a pragmatic approach (mixed methods study). This included quantitative and qualitative assessment to establish the clinical impact, costs and barriers for adoption of Home Hospitalization/Early Discharge, both jointly and separately, throughout the process of its implementation in one healthcare sector of the city of Barcelona from 2006 to 2015.

## Theory and methods

### Study design and ethics

This study was planned as a prospective, pragmatic assessment of the implementation of Home Hospitalization/Early Discharge as a mainstream home-based service delivered by the Integrated Care Unit at Hospital Clínic. The choice for a pragmatic approach, as opposed to an explanatory design, was guided by the ambition of generalizing the results in routine practices. In this regard, pragmatic designs are superior to explanatory ones to assess if an intervention works in real life [20]. Our study includes data from a 10-year period, from January 2006, when the program was first deployed into daily practice, to December 2015. The Ethical Committee for Human Research at Hospital Clinic approved the study and all participants signed an informed consent previous to any procedure. The program has been registered (ClinicalTrials.gov: NCT03130283) for the current report, as part of the analysis for regional deployment of the service.

### Patient eligibility

All acute or exacerbated chronic patients as well as surgical patients fulfilling criteria for admission in the Hospital Clinic were considered as potential candidates for Home Hospitalization/Early Discharge. In all instances, patients included in the study were recruited either at the Emergency Department (Home Hospitalization) or in a general Hospitalization Ward (Early Discharge).

Individuals were included in the study if: i) living in his/her house within the healthcare sector; ii) having a carer during 24h per day; iii) having phone at home; and, iv) signing written acceptance to participate in the study. Patients were excluded if: i) living in a nursing home; ii) high risk of severe clinical deterioration not treatable at home, as assessed by best medical judgment iii) admission in a short stay unit; iv) severe psychiatric disorder, and, v) insufficient manpower of the professional team running the program for additional admissions to Home Hospitalization/Early Discharge. Patients not meeting inclusion criteria were admitted to the acute care hospital wards (see on line supplement for complementary material).

### Definition of Home Hospitalization

We defined Home Hospitalization/Early Discharge as a service providing acute, home-based, short-term complex interventions aiming at substituting conventional hospitalization fully (Home Hospitalization) [21,22] or partially (Early Discharge) [23]. The service was delivered by trained hospital personnel for a period of time that was not longer than the expected length of hospital stay for the patient’s diagnostic related groups involved [24]. The Hospital retained the entire clinical, fiscal and legal responsibilities. Virtual beds were used to support required administrative and clinical processes.

The technological platform supported core service functionalities: (i) patient-centered adaptive case management [25]; (ii) enhanced patient accessibility (support center), and (iii) enhanced coordination of professionals across healthcare tiers after discharge, as reported in detail in [25] and the on-line supplementary material.

### Characteristics of the Home Hospitalization/Early Discharge program

The Home Hospitalization/Early Discharge program and how health professionals provided it to patients is described in detail in the on-line supplementary material.

#### Assessment of candidates

Patient evaluation at baseline was done by the professionals of the Home Hospitalization/Early Discharge team using standardized questionnaires [16,24,26,27,28,29]. The professionals also reviewed the patient’s electronic medical record.

#### Home-based intervention

The intervention was composed of a set of well standardized actions. The way these actions were applied to each patient depended on his/her predefined health conditions and social circumstances.

The first home visit was done within 24 h following admission to the programme. Moreover, daily home visits (~40 min duration) were carried out by a registered nurse with special training. This nurse was qualified to decide on performance of some complementary tests at home, namely: arterial blood gases, blood analytics, sputum culture, forced spirometry, etc…. He/she could remotely access the patient electronic medical record and contact the responsible physician by means of a laptop and a dedicated application. A physician’s home visit could be decided upon patient’s clinical evolution.

If during the Home Hospitalization/Early Discharge period, the patient needed a specific hospital visit (e.g. due to an unexpected event or a required testing), this situation did not imply withdrawal from the program unless in-hospital admission was mandatory. The entire Home Hospitalization/Early Discharge team met daily at the Integrated Care Unit to share information, discuss on patients’ evolution and decide on patients to be discharged.

Upon discharge from the programme, two complementary reports were prepared (one by the physician and the other one by the registered nurse with special training). These reports were uploaded into the patient electronic medical record that, in turn, forwarded them to the regional shared electronic health record. The physician and the registered nurse with special training made jointly the discharge visit at patient’s home.

#### Outcomes

Main study clinical outcomes assessed at 30-day post-discharge were: i) Hospital readmissions; ii) Emergency Department visits; iii) Mortality; iv) length of stay; and v) Cost of care. For patient’s and carers’ satisfaction we used a standardized questionnaire [30]. Qualitative data regarding the adoption of technology in the context of the service was assessed by the Method for ASsessment of Telemedicine applications [31]. This was complemented by qualitative insights obtained from the quality improvement sessions of the Integrated Care Unit. These sessions included regular internal meetings among the Integrated Care Unit team but also meetings with other hospital’s teams and departments that referred patients to the unit and also with professionals involved in the transition to community following discharge.

### Statistical analysis

Qualitative variables were expressed as absolute frequencies and percentages, whereas quantitative variables were summarized as mean and standard deviation in the case of normally distributed or median percentiles 25 and 75 otherwise. Student t-test or Mann-Whitney U test were used for comparisons of independent groups. Chi2 test or Fisher’s exact test were used for the comparison of proportions. Multivariate logistic regression analyses were performed to explore associations between selected covariates and main outcomes used as dependent variables in the models. Selection for potential covariates to be included in the logistic analyses was done exploring univariate associations between candidate variables and main study outcomes. Inclusion criteria for covariates were established at a p-value below 0.2. All statistical analyses were performed using SPSS statistical software package for Windows (version 20, IBM, Chicago, USA). All analyses were based on the bilateral hypothesis (two-tailed) with statistical significance below 0.05. Only direct healthcare costs, expressed in constant euros of 2015, were included in the economic analysis.

## Results

**Figure 1:**
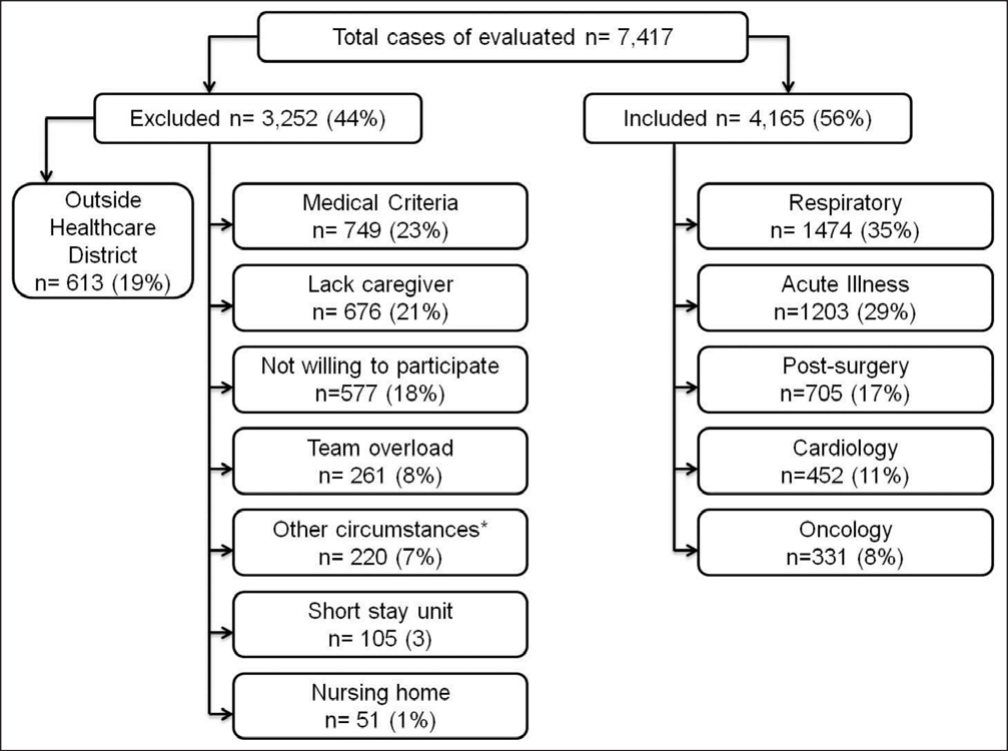
Flow chart of the study. *Other circumstances (marginal social condition, severe psychiatric disorder and lack of health insurance). See on line supplement for classification of diagnoses and clustering by diagnostic group.

Figure 1 depicts the study flow. Characteristics of the Home Hospitalization/Early Discharge patients and most common main diagnoses are indicated in Table 1 and Table 1S, respectively.

**Table 1:** General characteristics of discharges.

Episodes, n	Total n = 4,165	HH n = 2,529 (61%)	ED n = 1,636 (39%)	p value	

**Socio-Demographic** *(patient interview)*					

Age (yrs), m ± SD	71 ± 15	73 ± 15	69 ± 14	<0.001	*
Women, m ± SD	1,563(38)	1,011(40)	552(34)	<0.001	^¥^

**Health Care resources** *(patient interview and clinical record)*					

Home care prior admision					
Primary Care, n(%)	393(9)	278(11)	115(7)	<0.001	^¥^
Palliative care, n(%)	58(1)	38(2)	20(1)	0.440	^¥^
Hospital, n(%)	107(3)	80(3)	27(2)	0.002	^¥^
Without any suppot at home, n(%)	3,607(87)	2,133(84)	1,474(90)	<0.001	^¥^
Hospital resources in previous 12 m					
Visits to ER, (Median, P_25_–P_75_) m ± SD	0(0–1) 1 ± 1	0(0–1) 0.8 ± 1	0(0–1) 0.7 ± 1	<0.001	*
Hospital admissions, (Median, P_25_–P_75_)	0(0–1) 1 ± 1	0(0–1) 0.8 ± 1	0(0–1) 0.7 ± 1	0.001	*

**Chronic conditions** *(clinical record)*					

>1 chronic conditions, n(%)	3,184(76)	1 958(77)	1,226(75)	0.043	^¥^
n° of comorbidities, m ± SD	4 ± 3	4 ± 3	4 ± 2	<0.001	*
Charlson Index, m ± SD	4 ± 3	5 ± 3	4 ± 3	<0.001	*
**Main Diagnóstic Group, n(%)**					
Respiratory n(%)	1,474(35)	1,018(40)	456(28)	<0.001	*
Cardiology n(%)	1,203(29)	367(15)	85(5)	<0.001	*
Post-surgery n(%)	705(17)	20(1)	695(42)	<0.001	*
Oncology n(%)	452(11)	260(10)	71(4)	<0.001	*
Acute illness n(%)	331(8)	864(34)	339(21)	<0.001	*

**Risk factors and treatments** *(patient iterview and clinic record)*					

Smoking (pack/yr) (Median, P_25_–P_75_)	(n = 1,651) 37(15–60)	(n = 948) 36(14–60)	(n = 703) 37(15–60)	0.917	*
Active smoker, n(%)	557(13)	303(12)	254(16)	0.001	^¥^
Ex-smoker, n(%)	1,898(46)	1,151(46)	747(46)	0.948	^¥^
BMI, m ± SD	(n = 3,087) 27 ± 5	(n = 1,841) 27 ± 5	(n = 1,246) 27 ± 5	0.593	*
Sedentary lifestyle, n(%)	1,038(25)	739(30)	299(19)	<0.001	
Treatment prior admission					
Oxygen therapy at home, n(%)	453(11)	300(12)	153(9)	0.156	
CPAP/BIPAP, n(%)	171(4)	105(4)	66(4)	0.298	
n° pills/day, m ± SD	6 ± 5	6 ± 5	5 ± 4	<0.001	*
n° inhalations/day, m ± SD	1.9 ± 3.3	2.2 ± 3.4	1.6 ± 3.1	<0.001	*
Knowledge of chronic disease					
Name of disease, n(%)	(n = 3,846) 2,049(53)	n = 2,332 1,210(52)	n = 1,532 839(55)	0.080	^¥^
Alarm signs + had an action plan, n(%)	(n = 3,816) 1,492(39)	n = 2,325 890(38)	n = 1,521 602(40)	0.419	^¥^
Difficulty in treatment delivery, n(%)	(n = 3,816) 274(7)	n = 2,314 185(8)	n = 1,502 89(6)	0.02	^¥^

**Patient’s dependence factors** *(self-administrated questionnaires)*					

Quality of life (SF-36)					
Physical status, m ± SD	33 ± 13	32 ± 13	34 ± 12	0.053	*
Mental status, m ± SD	41 ± 16	41 ± 16	42 ± 15	0.097	*
Daily life activities (Barthel Index), (Median, P_25_–P_75_)	(n = 4,107) 89 ± 21 100(85–100)	(n = 2,501) 87 ± 23 100(85–100)	(n = 1,606) 91 ± 18 100(90–100)	<0.001	*
Mild dependence, n(%)	921(22)	597(24)	324(20)	0.006	^¥^
Independent, n(%)	2,487(61)	1,427(57)	1,060(66)	<0.001	^¥^

**Legend:** Data are expressed as mean ± standard deviation for quantitative variables and number (percentage) for discrete variables. It is expressed as median (25–75th Percentile) in quantitative variables with extreme values. ^¥^Chi2 test or Fisher’s exact test were used for the comparison of proportions. Indicates statistical significant differences between the two groups. *The Mann-Whitmey U test was used for variables not normally distributed. Home Hospitalization (HH), Early Discharge (ED), Emergency Room Department (ER), Body Mass Index (BMI), Continuous Positive Airway Pressure (CPAP), Bilevel positive airway pressure (BIPAP), The Short Form (36) Health Survey (SF-36).

Seventy-six percent of Home Hospitalization/Early Discharge admissions had at least one chronic condition and the Charlson Index adjusted by age was 4 ± 3. Full hospital avoidance (Home Hospitalization) was achieved in 61% of the cases (n = 2,529), 69% of them were admitted to Home Hospitalization directly from the Emergency Department and 31% from a general hospitalization ward. It is of note that 42% of the patients admitted in Early Discharge (n = 1,636) were post-surgical cases.

Throughout the study period there was a steady increase in the complexity of the patients indicated by: (i) Number of co-morbid conditions, from 3 ± 2 to 6 ± 2; and (ii) Need for intravenous therapies (p < 0.001) and complex dressings & care (p = 0.002). Differences between Home Hospitalization patients and those included in Early Discharge are depicted in Tables 1 and 1S.

### Results of the intervention

As shown in Table 2, the intervention reduced the in-hospital stay to one day, as a median. Despite the increase in patient complexity over time, the mortality rate did not increase (2%) at 30 days post discharge.

**Table 2:** Comparisons between the study group (Home Hospitalization/Early Discharge).

Variable	Total	HH	ED	p value	

n° discharge	4,165	2,529	1,636		

**In-Hospital stay, days**					
Hospital stay (Median, P_25_–P_75_)	1(0–3)	0(0–1)	4(2–7)	<0.001	*
**Home stay, days**					
Home stay (Median, P_25_–P_75_)	6(5–7)	6(5–7)	5(4–7)	<0.001	*
**Total length of stay, days**					
In-hospital + Home (Median, P_25_–P_75_)	7(6–10)	6(5–8)	10(8–14)	<0.001	*
**Use of resources during HH/ED**					
Number of Physician visits, m ± SD	1 ± 0.5	0.85 ± 0.46	0.85 ± 0.43	0.735	*
Number of nurse visits, m ± SD	7 ± 3	6.6 ± 2.8	6.5 ± 3.6	<0.001	*
Number phone call to the patient, m ± SD	2 ± 1	2 ± 1	2 ± 1	0.903	
Emergency Room visits, n(%)	68(2)	45(2)	23(1)	0.353	^¥^
In-Hospital re-admissions, n(%)	201(5)	127(5)	74(4)	0.463	^¥^
**Outcomes at 30 days after HH/ED discharge**					
Emergency Room visits, n(%)	311(7)	181(7)	130(8)	0.448	^¥^
Hospital admissions, n(%)	461(11)	288(11)	173(11)	0.441	^¥^
**Mortality**					
During episode, n(%)	12(0.3)	10(0 4)	2(0.1)	0.108	^¥^
During 30 days post discharge, n(%)	94(2)	73(3)	21(1)	0.001	^¥^
**Transitional Care after HH/ED discharge**					
Primary care n(%)	3,527(85)	2,077(82)	1,450(89)	<0.001	*
Palliative care n(%)	226(5)	156(6)	70(4)	<0.001	*
Hospital n(%)	292(7)	215(8)	77(5)	<0.001	*

**Legend:** Data are expressed as mean ± standard deviation for quantitative variables and number (percentage) for discrete variables. It is expressed as median (25–75th Percentile) in quantitative variables showing extreme values. ^¥^Chi2 test or Fisher’s exact test were used for the comparison of proportions. Indicates statistical significant differences between the two groups. *The Mann-Whitmey U test was used for variables not normally distributed. Home Hospitalization (HH), Early Discharge (ED).

The in-hospital days progressively decreased from 2 (1–4) to 1(1–3) during the period of the study. Moreover, the rate of hospital re-admissions also steadily decreased (from 13% to 11%), and a similar trend was observed for the rate of emergency room visits (from 7% to 6%). However, the number of home nurse visits per year increased steadily (p < 0.001). To ensure a safe transition after Home Hospitalization/Early Discharge, patients were allocated to the appropriate level of care and program according to his/her individual action plan.

Applying logistic regression analyses to the results of early re-admissions at 30 days after Home Hospitalization/Early Discharge (Table 2S), we identified covariates associated to high risk for early re-admissions (Table 3). Briefly: (i) Charlson index ≥3, (ii) History of previous hospital admissions, (iii) Specific diagnoses such as heart failure and cancer; and, (iv) High degree of dependency were associated with increased rate of early re-admission. Moreover, cancer and sedentariness were associated with mortality at 30 days after discharge.

**Table 3:** Unadjusted and Adjusted Regression Model, 30 days after Home Hospitalization/Early Discharge.

	HH/ED		HH (<24 h)	ED (>24 h)

	Unadjusted ORs (CI95%) p value	Adjusted Ors (CI95%) p value	Adjusted Ors (CI95%) p value	Adjusted Ors (CI95%) p value

Age (yrs)	0.765(0.622–0.940) p = 0.011			
Gender (women)	0.765(0.622–0.940) p = 0.011			
Charlson Index ≥3 points	3.145(2.185–4.527) p = 0.001	3.247(1.723–5.503) p < 0.001	2.945(1.160–7.474) p = 0.023	3.448(1.323–8.900) p = 0.011
n comorbidities >1	1.480(1.151–1.903) p = 0.002			
Daily physical activity	0.678(0.549–0.837) p < 0.001			
Dependency (Barthel Index)	1.519(1.249–1.849) p < 0.001	1.648(1.190–2.281) p = 0.003	1.751(1.145–2.678) p = 0.010	
Previous Hospital admission (12 m)	1.285(1.209–1.365) p < 0.001	1.218(1.098–2.010) p = <0.001	1.308(1.138–1.503) p < 0.001	
LTOT	1.414(1.066–1.877) p = 0.016			
Heart Failure	2.092(1.616–2.707) p < 0.001	2.000(1.325–3.020) p = 0.001	2.126(1.324–3.414) p = 0.002	
Post-surgery	0.755(0.571–0.989) p = 0.048			
Oncology	1.984(1.477–2.665) p < 0.001	2.510(1.152–5.029) p = 0.009		
SF-36 physical domain	0.977(0.967–0.998) p < 0.001			
n Pills/day	1.073(1.052–10.96) p < 0.001			1.070(1.010–1.132) p = 0.021
Palliative care	1.920(0.988–3.729) p = 0.052			5.525(1.025–10.25) p = 0.006
Intervention (HH/ED)	0.920(0.754–1.124) p = 0.414		NA	NA

**Legend:** Long Term Oxygen Therapy (LTOT); The Short Form (36) Health Survey (SF-36); Home Hospitalization (HH), Early Discharge (ED).

Evaluation applying the Method for ASsessment of Telemedicine applications (Table 4S) indicated safety of the intervention, as well as high degree of users’ satisfaction including patients, careers and health professionals. Adequate skills of health professionals providing Home Hospitalization/Early Discharge and proper coordination with community-based services were identified as key requirements of the setting (see professional profile in the on-line supplementary material for detailed information).

The monthly meetings of the members of the Integrated Care Unit were a source of information about those aspects related to the practicalities of the provision of the services and the coordination with other services (other hospital departments, external service providers –e.g. companies providing respiratory equipment therapies, home rehabilitation services…, and community care). The ultimate goal of these meetings was to improve the quality of the service and expand the portfolio of the Integrated Care Unit to better response to the demands expressed by other hospital departments and the community care. This team-based quality improvement strategy contained elements that were useful for adapting the services of the unit to changing needs and supported the analysis of the quantitative and qualitative data done in the present study.

### Economic analysis

When the Home Hospitalization/Early Discharge service was first deployed in 2006, a flat rate reimbursement scheme of 918/discharge was applied whereas the cost of conventional hospitalization was 2,879€/discharge. Consequently, the program reduced healthcare costs due to reduction of in-hospital stay (Table 2) with impact in the overall health system. Moreover, a detailed cost analysis (Table 3S), indicated a positive net balance for the providers despite the program was applied to patients whose complexity increased during the period of the study.

## Discussion

### Main findings and determinants of the study outcomes

The current report describes the pragmatic assessment of Home Hospitalization/Early Discharge deployment over a period of 10 years in one of the healthcare sectors of the city of Barcelona. The results demonstrate safety and effectiveness with high level of user’s acceptance and health value generation of the approach. It is of note that 82% of eligible cases to whom the service was offered accepted the option. Moreover, a high level of satisfaction was reported by 98% of both professionals and patients after Home Hospitalization/Early Discharge. Overall, the main findings of this 10 year pragmatic assessment study are summarized below.

Firstly, at hospital level, Home Hospitalization/Early Discharge has demonstrated high potential for scalability for both, acute medical/surgical, and chronic patients admitted in the Hospital and ensured a safe transitional care after Home Hospitalization/Early Discharge. Moreover, during the deployment of the services, any issues raised regarding the clinical process, logistics and organization-ethics were solved satisfactorily (despite the progressive increase in case complexity observed during the study). The implementation of stratification strategies seems to be an interesting option to objectively address the tuning of services to patients and this needs further exploration.

The study consolidated the Home Hospitalization/Early Discharge service as the first choice for the described profiles of patients when admitted in the Emergency Department. The number of virtual beds available for the service increased from 12 virtual beds/day (2006) to 36 (2016) with plans for further increases over a three-year period. Moreover, the current Home Hospitalization/Early Discharge service is active on a 24 × 7 basis all year round. It enjoys a strong economic incentive since the change of the reimbursement scheme that made the fee equal to conventional hospitalization.

Secondly, this study has looked into the challenge posed by the transition of patients from hospital to the community following discharge from home hospitalisation [32,33,34,35,36,37]. Two distinct scopes of action for the hospital-based home-hospitalisation teams in the community seem to emerge: The first scope of action comprises the role of home hospitalisation in the transition from hospital to community for those patients that can be safely managed by the primary care teams. This scope of action is well established. However, there is still room to investigate whether hospital-based teams can provide additional support to primary care teams in retaining patients in the community. A second scope of action relates to the role that home-hospitalisation can play in the transition from hospital to community in more complex patients, that is those still requiring frequent specialist follow-up and interventions. More research is needed to characterise this scope of action, including: identification of eligible patients (applying smarter criteria in addition to risk stratification tools) and composition of the home-hospitalisation teams (registered nurses with specific training but also mix of specialists and primary care professionals willing to work overcoming traditional organisational boundaries and jurisdictions) [12,32,33,34,38,39,40,41].

Thirdly, at service commissioning level, the study identified the key factors determining transferability of Home Hospitalization/Early Discharge to other sites, as extensively reported elsewhere [16]. The comparative analysis of the deployment in Barcelona, Norway and Greece indicated that: (i) High quality specialized professionals supporting the hospital outreached service [42]; and, (ii) Close coordination between specialized professionals and community-based services constitute two equally important requirements to ensure service adoption.

### Comparison with the literature

The meta-analysis carried out by Caplan et al [2] including 61 worldwide randomized controlled trials reported maturity and health value generation of Home Hospitalization/Early Discharge programs. But, the most recent Cochrane review [7,23] concludes that Home Hospitalization/Early Discharge is indicated for selected groups of elderly patients adding a degree of imprecision to the results for the main outcomes. Moreover, efficient care coordination after Home Hospitalization/Early Discharge is relevant, but it is often an unmet need.

Our results are aligned with Caplan’s report l [2], but our study shows some distinctive traits with regard to other reported experiences. Thus, we describe the factors that modulate Home Hospitalization/Early Discharge deployment in a real world scenario with no constraints in terms of age and diagnostic criteria. Moreover, Home Hospitalization and Early Discharge were assessed, jointly and separately. Stepwise service adaptive changes required by the progressive increase in patients’ complexity are described in detail in the online supplementary material. The use of the Method for ASsessment of Telemedicine applications [31] showed relevant outcomes in all seven dimensions considered.

Our study emphasizes the importance of the interplay between highly specialized medical care and community-based services in generating health value by transferring service complexities from hospital to home through appropriate portfolio services [39]. This assumes collaborative work across healthcare levels, an aspect that implies in itself a number of challenges. In addressing these challenges, our study supports the need to redefine the roles of professionals and their scopes of action in innovative ways. Examples of such novel approaches could be: hospital based specialists moving into home-hospitalisation teams that manage complex patients in the community; empowered hospital and primary care nurses mastering advanced care practices that could intervene beyond their traditional professional competences; or, mixed teams of specialists, primary care professionals and empowered nurses organised to operate as a self-managed health unit in the territory [43].

### Study limitations and strengths

We acknowledge that the study design adopted in the current report may show inherent weaknesses if the aim was to generate strong evidence on efficacy of Home Hospitalization/Early Discharge. The implementation research approach [44] adopted in the report has provided novel valuable information on key factors modulating both scalability and transferability of this type of services. We believe that the current assessment of Home Hospitalization/Early Discharge deployment, with a 10-year follow-up period, is likely the most appropriate design for the purposes envisaged in the study. Other traits such as the precise definition of Home Hospitalization/Early Discharge adopted and the structured services workflows represent strengths of the study because they avoid ambiguities in the use of the term and facilitate comparability with other experiences.

### Future research perspectives

We identify several major areas deserving further research in this field in order to improve both outcomes and sustainability of Home Hospitalization/Early Discharge services. Firstly, novel developments in the area of dynamic risk assessment and clinical stratification of patients eligible for this type of services following the strategies proposed in [45].

The second area refers to research on applicability of bundled-based innovative business models following the proposals reported in [16,46]. The approach should generate incentives for hospital providers as well as stimulate care coordination across healthcare tiers. Finally, the third area of interest refers to research on novel methods to enhance workforce competences preparation for integrated care services. No need to mention that structured, but flexible, service workflow definition is needed in other to facilitate comparability, as well as ensure proper evaluation, among deployment of Home Hospitalization/Early Discharge experiences [47].

## Conclusions

The results support effectiveness of the approach proposed in the current study wherein Home Hospitalization/Early Discharge represents a highly efficient component of the care coordination ecosystem bridging hospital-based specialized care with community services.

## Additional File

The additional file for this article can be found as follows:

10.5334/ijic.3431.s1 Appendix
