# Supplementary material for: Implementation of Home Hospitalization and Early Discharge as an Integrated Care Service: A Ten Years Pragmatic Assessment
Source: Int J Integr Care. 2018 May 16;18(2):12. doi: 10.5334/ijic.3431 (PMC6095082; doi:10.5334/ijic.3431)
Supplement: Appendix [file ijic-18-2-3431-s1.pdf]

(1)MATERIAL AND METHODS

(1.1)Healthcare system

The urban healthcare sector wherein deployment of Home Hospitalization/Early Discharge was carried out includes the Hospital Clinic as reference center, two general hospitals and 19 primary care centers run by different healthcare providers.

(1.2)ICT-support for HH/ED

(1.2.1)Patient-centered management *(adaptive case management)[1].* Cover a variety of stages: *i)* case identification; *ii)* case evaluation, *iii)* work plan definition; *iv)* follow-up event handling; and, *v)* discharge. Case identification refers to the patient's information required at the entry point. Case evaluation includes assessment of the patient to determine his/her eligibility for Home Hospitalization/Early Discharge and to capture the information required for the next step, work plan definition. The work plan consists on a set of both timed and non-timed tasks, led by a team of healthcare professionals, aligned with the aims of the Home Hospitalization/Early Discharge. Follow-up and event handling correspond to the execution of the working plan. It is of note that the continuous patient's assessment every day may lead to changes in the working plan triggered by unexpected events. Finally, the case is prepared for his/her discharge from the Home Hospitalization/Early Discharge.

(1.2.2)Enhanced patient accessibility *(support center).* It is one of the main access points for patients, caregivers and professionals and it plays a central role for three specific functions: *(i)* patient's triage, *(ii)* promoting patient self-management, and, *(iii)* management of remote monitoring. The tele-operator in the Support Centre receives different types of requests (health issues, administrative problems and social support requests) that are solved by health professionals of the Integrated Care Unit. The system is operational only for target patients that are managed by programs following well-standardized interventions and providing a patient-centered care. Home Hospitalization/Early Discharge is one of the program.

(1.3)Characteristics of the HH/ED program

(1.3.1)Structure

It was based at the Integrated Care Unit, a transversal organization created in 2006 to foster coordinated care patients through the deployment of Integrated Care Services. It is a transversal department under the Medical and Nursing directions of the Hospital Clinic. Its mission was to provide support to the different medical specialties and to facilitate the bridging between the hospital-based specialized care and the community after hospital discharge.

(1.3.2)Professional profile

The central Home Hospitalization/Early Discharge program was carried out by specialized hospital teams. It was composed by one internal medicine physician, four full-time Registered Nurses with special training, plus one part-time Registered Nurse with special training, two part-time administrative officers with special training attending the call center as well as a PhD, RN coordinator.

Seventy percent of Registered Nurses with special training are nurse plus Master and all of them have worked at least 15 years at the hospital (Intensive Care Unit or

Emergency room) before moving to the Integrated Care Unit. The internal medicine physician has worked 10 years at the hospital (Internal Medicine ward, Emergency Room and Intensive Care Unit). All of professionals needed at least 3 months in our Unit, for additional training.

(1.3.4)Organizational issues

The Home Hospitalization/Early Discharge program was initiated early 2006, as a prolongation of previous pilot experiences with chronic respiratory patients[2–5]. The real deployment was initiated in patients with chronic respiratory disease and heart failure because of previous experience. Several hospital departments progressively joined the program diversifying patients' characteristics and worked with the Home Hospitalization/Early Discharge team in a joint protocol using the clinical guidelines of each specialty.

The program was open from Monday to Sunday, between 8.00 am and 09:00 pm. The period for patients' inclusion in the Home Hospitalization/Early Discharge program was from 09:00 am to 05:00 pm, from Monday to Friday, wherein the internal medicine physician was present at the Integrated Care Unit. Incidences occurring from 05:00 to 09:00 pm and during the weekend were attended by the Registered Nurses with special training that had access to doctors on duty. For incidences between 09:00 pm to 08:00 am, patients could either leave a voice message or contact directly one of the specialists on duty through the call center.

(1.4)Intervention

(1.4.1)Patient eligibility and decision process for inclusion

Patient eligibility was assessed in the emergency room (Home Hospitalization) or in the general ward (Early Discharge). Candidates were identified by the physicians of the respective units and posterior contact with the Home Hospitalization/Early Discharge team (physician and case manager (Registered Nurses with special training), who verified the patient's eligibility for Home Hospitalization/Early Discharge using a standard protocol at the time of enrollment. The time between the patient identification and patient assessment by the Home Hospitalization/Early Discharge team did not exceed 2 hours.

(1.4.2)Assessment

The essential information was grouped into five dimensions[6], namely: *i)* socio-demographics [7] *ii)* health team and system related factors [7]; *iii)* characteristics of patient's chronic conditions and Primary diagnosis[8–11] ; *iv)* risk factors and treatment[12] and, *v)* patient dependence factors (SF-36[13], Barthel Index[14]) **(Tables 1, 1S, 2, 2S, 4S)**. Additional information was obtained from the patient electronic health records following the current legislation on access and confidentiality of the clinical data. Assessment and follow-up were carried out using the Information and Communication Technology platform described in detail in[15].Thirty days after discharge, patients and caregivers were administered a questionnaire on satisfaction with the Home Hospitalization/Early Discharge program via telephone[7] .

(1.4.3)From hospital to home

Patients were transferred to home by ambulance on the same day of the evaluation after installing at home the equipment needed during Home Hospitalization/Early Discharge period. The program provides home equipment (oxygen, non invasive

mechanical ventilation, nebulizer, pump, glucometer, etc) and offers the possibility to performing some tests at home (forced spirometry, etc), intravenous treatment (continue by pump or discontinue), measurements (blood or fluids testing), dressing and drains. Pharmacological treatment at home was prescribed by the Home Hospitalization/Early Discharge physician and prepared by the hospital pharmacy. The time elapsed between the end of the patient's evaluation and his/her arrival at home with all logistics ready for Home Hospitalization/Early Discharge did not exceed 4 hours.

(1.4.4)Home intervention

At the time of arrival at home, a telephone call to the patient was made by the Registered Nurse with special training. All patients received basic therapeutic educational material. The interventions were planned following the international guidelines for each diagnostic group. The program was conducted with a patient-oriented approach wherein management of co-morbid conditions and adherence to therapy had central roles. The home visits included: *i)* assessment of patient clinical status; *ii)* control of co-morbid conditions; *iii)* revision of the treatment plan including dressings and administration of intravenous treatment if prescribed; *iv)* reinforcement of therapeutic education and adherence; *v)* checking of the equipment installed at the patient's home; and, *vi)* assessment of environmental conditions. Remote patient self-monitoring (pulse oximeter, spirometer, scale and glucometer) were available to incorporate in the individualized plan.

The specific home-based interventions during the Home Hospitalization/Early Discharge period were: *(i)* intravenous therapies in 54%; *ii)* peripheral blood sampling for biological analysis in 53%, *iii)* transient oxygen therapy, 39% of the cases and nebulizer therapy in 24%; *(iv)* complex dressings and care in 21%; *(v)* arterial respiratory blood gas measurements in 13%; *(vi);* and; *(vii)* forced spirometry in 10% of the cases. Discharge from Home Hospitalization/Early Discharge could be related to improvement, cure, hospital admission or death.

(1.4.5)Enhanced coordination of professionals across healthcare tiers

Hospital infrastructure: department ward, emergency room area and laboratories. One physician of each specialized department provided support to the Home Hospitalization/Early Discharge team, if needed

Services providers:

a) During Home Hospitalization/Early Discharge: Companies providing respiratory equipment therapies, if needed.
b) After Home Hospitalization/Early discharge. Primary care and palliative care team, the complex frail patient program and the outpatient clinic from the hospital to ensure transitional care. All services were coordinated by the Integrated Care Unit.

(2)RESULTS

(2.1)Classification of diagnoses and clustering by diagnostic groups

As part of the disease classification process, up to 850 different ICD-9-CM coding[9] were included in the Home Hospitalization/Early Discharge, as main or secondary diagnoses. It is of note that 455 of them (54%) were chronic conditions. All 850 ICD-9 entities were grouped by biological systems[11]. The allocation of each admission into a specific subgroup was performed considering the principal diagnosis at admission.

Patients were grouped in five diagnostic groups. ICD9 -CM coding corresponding to each of these groups is indicated below:

Respiratory: 491.1, 491.20, 491.21, 491.22, 491.9, 496, 492.0, 492.8, 491.03, 493.02, 493,01, 493,91, 493.92, 516, 516.8, 518.81, 277.02, 494.1, 519.0, 519.9, 519.8, 599.0, 486, 486.6, 507.0, 502.0, 786.3, 786.39, 518.89, 516.31.

Acute illness: 466, 486, 488, 599, 590.1, 682, 009.0, 513, 012.0, 415.1, 421.0, 560, 338.3, 451.89, 453.4, 507.0, 572.0, 576.1, 285, 601.0, 682.0, 682.6, 682.9, 707.1, 733.13, 733.19, 780.6, 999.31, 338.3, 787.91, 287.4, 711.9, 494.1, 487.

Post-surgery: 414, 424, V58.73, v58.78, V49.72, 733.15, 444, 553, 730, 250.7, 416.8, 427.31, 440.02, 442.0, 445.5, 958.3, v49.7, v58.7, 86.83, 715.96, 415, 733.14, v69.09, 958.3, 688.83, 998.59, 32.3, 60.5, 51,23, 569.81, 746.4, 569.81, 746.4, 440, 576.1, 425.4, 394.2, 396.3, 579.3, 604.9, v66, v55.3, v84.15, 996.76, 580.9, 576.22, 580.9, 542, 442, 997.49, 730, 566, 423.9, 395.0, 557, 414.0, 553.

Cardiology: 410, 448, 428-429, 446.5, 402, 414.01, 416, 404.91, 424.1, 427, 398.91.

Oncology: 486, 288.03, 488, 484.8, 513, 516, 560, 009.0, 053.9, 287.4, 288.0, 388.3, 415.1, 419.21, 543.2, 453.4, 484.6, 507.0, 576.1, 590.1, 682.0, 682.6, 682.9, 729.30, 780.6, 786.3, 787.91, 999.31, 225.2, 999.31, 787.91, 196.8, 287.4, 711.9, 53.9, 338.3.

(2.2)HH/ED readmissions

Comparisons between patients re-admitted within 30 days after Home Hospitalization/Early Discharge and those who did not, allowed identification of several variables showing a significant associations with early re-admissions, namely: men (p=0.001); older age (p<0.001), ex-smoker (p<0.001); number of co-morbidities (p=0.006), Charlson Index[8] (ref) (p<0.001); Cardiology (p<0.001), Oncology (p<0.001), sedentary lifestyle (p<0.001), low health-related quality of life (SF-36[13]; physical, p=0,007) history of previous use of hospital resources, such as Emergency Department visits (p<0.001) and hospital admissions (p<0.001) and were follow up by palliative home care programs before Home Hospitalization/Early Discharge admissions (p=0.050).

We observed that patients re-admitted within 30 days after Home Hospitalization/Early Discharge required more intravenous treatment (p<0.001), blood test (p=0.007) and more nebulizer therapy (p=0.030) at home **(Table 2S).** The analysis was evaluated for the entire study group, as well as for Home Hospitalization and Early Discharge separately.

(2.3)Analysis of other MAST dimensions[16] (Table 4S)

Patients' perspectives - The patient's rejection rate for inclusion to the program was kept at a level of 18% throughout the study period. It is of note, however, that both patients and caregivers accepting to participate showed a high rate of satisfaction: 99 % of the subjects reported that the treatment received was very good. Moreover, 90% of the patients and 94% of the caregivers stated that they would repeat the experience if needed.

Professionals' perspectives - Initial common resistances to implementation from both Hospital and Primary Care staff markedly decreased over time. Hospital-based specialists fear to lose beds; whereas primary care professionals look at the Home

Hospitalization/Early Discharge program as intrusive. In contrast, professionals of the Home Hospitalization/Early Discharge team showed high degree of satisfaction throughout the deployment period.

Organizational and regulatory aspects –The re-engineering process initiated in 2006 through an innovative program fostering collaboration between specialized and primary care and the creation of the Integrated Care Unit facilitated the deployment of Home Hospitalization/Early Discharge, as explained in detail in[15]. The entire integrated care program deployed in NEXES enforced the bridging between hospital and community care throughout the study period.

Technologies – From the Information and Communication Technology standpoint, the four major lessons learnt during the study period were: *i)* interoperability at a health system level, across levels of care and among providers, is a must to optimize the program; *ii)* remote monitoring used by professionals visiting patient-home showed high efficacy and it was a source of cost-containment; *iii)* patient self-monitoring showed limited potential because of two main factors: the acute condition of the patient and the short available learning period, and, *iv)* interactive tools such as videoconference and the patient personal health folder[17] seem to be useful to reinforce remote support, but we were unable to optimize these two Information and Communication tools during the project lifetime.

(2.4)Economic analysis

The economic analysis included the impact of Home Hospitalization/Early Discharge on both healthcare provider and at health system level.

The Home Hospitalization/Early Discharge program admitted between 303 and 559 patients per year over a ten-year period, from 2006 to 2015. **Table 3S** displays the costs of the different items and reimbursement per year.

The Home Hospitalization/Early Discharge was highly efficient when the economic analysis is done at health system level due to an average saving of 6 in-hospital days per patient. Indirect costs were not included because of: 1) the payer perspective adopted; 2) the fact that the need for an additional care was a criterion of exclusion of the program, and 3) because the majority of informal careers are retired spouses.

(3)Reference List

1. Cano I, Alonso A, Hernandez C, Burgos F, Barberan-Garcia A, Roldan J, Roca J (2015) An adaptive case management system to support integrated care services: Lessons learned from the NEXES project. J Biomed Inform 55: 11-22. S1532-0464(15)00045-3 [pii];10.1016/j.jbi.2015.02.011 [doi].

2. Casas A, Troosters T, Garcia-Aymerich J, Roca J, Hernandez C, Alonso A, del PF, de TP, Anto JM, Rodriguez-Roisin R, Decramer M (2006) Integrated care prevents hospitalisations for exacerbations in COPD patients. Eur Respir J 28: 123-130.

3. Garcia-Aymerich J, Hernandez C, Alonso A, Casas A, Rodriguez-Roisin R, Anto JM, Roca J (2007) Effects of an integrated care intervention on risk factors of COPD readmission. Respir Med 101: 1462-1469.

4. Hernandez C, Casas A, Escarrabill J, Alonso J, Puig-Junoy J, Farrero E, Vilagut G, Collvinent B, Rodriguez-Roisin R, Roca J (2003) Home hospitalisation of exacerbated chronic obstructive pulmonary disease patients. Eur Respir J 21: 58-67.

5. Puig-Junoy J, Casas A, Font-Planells J, Escarrabill J, Hernandez C, Alonso J, Farrero E, Vilagut G, Roca J (2007) The impact of home hospitalization on healthcare costs of exacerbations in COPD patients. Eur J Health Econ 8: 325-332. 10.1007/s10198-006-0029-y [doi].

6. WHO (2003) Adherence to Long-term Therapies. Evidence for Action.http://www.who.int/chp/knowledge/publications/adherence_report/en/index.html.

7. Generalitat de Catalunya. Departament de Sanitat i Seguretat social. Enquesta de salut de Catalunya (ESCA). 2002

8. Charlson ME, Pompei P, Ales KL, MacKenzie CR (1987) A new method of classifying prognostic comorbidity in longitudinal studies: development and validation. J Chronic Dis 40: 373-383.

9. Deyo RA, Cherkin DC, Ciol MA (1992) Adapting a clinical comorbidity index for use with ICD-9-CM administrative databases. J Clin Epidemiol 45: 613-619. 0895-4356(92)90133-8 [pii].

10. Fetter RB, Shin Y, Freeman JL, Averill RF, Thompson JD (1980) Case mix definition by diagnosis-related groups. Med Care 18: iii, 1-iii,53.

11. Hernandez C, Jansa M, Vidal M, Nunez M, Bertran MJ, Garcia-Aymerich J, Roca J (2009) The burden of chronic disorders on hospital admissions prompts the need for new modalities of care: a cross-sectional analysis in a tertiary hospital. QJM 102: 193-202.

12. Morisky DE, Green LW, Levine DM (1986) Concurrent and predictive validity of a self-reported measure of medication adherence. Med Care 24: 67-74.

13. Vilagut G, Ferrer M, Rajmil L, Rebollo P, Permanyer-Miralda G, Quintana JM, Santed R, Valderas JM, Ribera A, Domingo-Salvany A, Alonso J (2005) The Spanish version of the Short Form 36 Health Survey: a decade of experience and new developments. Gac Sanit 19: 135-150. 13074370 [pii].

14. Mahoney FI Barthel DW (2013) Functional evaluation: The Barthel Index. Md State Med J 1965 14:61-65 .

15. Hernandez C, Alonso A, Garcia-Aymerich J, Grimsmo A, Vontetsianos T, Garcia CF, Altes AG, Vogiatzis I, Garasen H, Pellise L, Wienhofen L, Cano I, Meya M, Moharra M, Martinez JI, Escarrabill J, Roca J (2015) Integrated care services: lessons learned from the deployment of the NEXES project. Int J Integr Care 15: e006.

16. Kidholm K, Ekeland AG, Jensen LK, Rasmussen J, Pedersen CD, Bowes A, Flottorp SA, Bech M (2012) A model for assessment of telemedicine applications: MAST. Int J Technol Assess Health Care 28: 44-51. S0266462311000638 [pii];10.1017/S0266462311000638 [doi].

17. Barberan-Garcia A, Vogiatzis I, Solberg HS, Vilaro J, Rodriguez DA, Garasen HM, Troosters T, Garcia-Aymerich J, Roca J (2014) Effects and barriers to deployment of telehealth wellness programs for chronic patients across 3 European countries. Respir Med 108: 628-637. S0954-6111(13)00492-7 [pii];10.1016/j.rmed.2013.12.006 [doi].

Table 1S. Main diagnoses of HH/ED admissions

Home Hospitalitation (n Total=2,529)	n(%)
COPD exacerbation (491.20, 491.21, 492.0, 492.8)	557(22)
Heart Failure/Complications of heart disease (428-429)	347(14)
Acute Bronchitis/ Respiratory system disease NOS/ Other pneumonopathy (466, 519.9,516)	350 (14)
Urinary tract infection (599)/Acute pyelonephritis (590.1)	280(11)
Pneumonia (486)	280(11)
Asthma exacerbation (491.03, 493.02)	110(4)
Bronchiectasis whit acute exacerbation (494.1)	78(3)
Fever and other physiologic disturbances of temperature regulation (780.6) (Cancer)	66(3)
Febril neutropenia (288.03)	63(2)
Limb cellulitis (682, 682.6)	57(2)
Acute venous embolism and thrombosis of deep vessels of lower extremity (453.4)	52(2)
Pneumonia. Aspiration (507)	45(2)
Pulmonary embolism and infarction (415.1)	44(2)
Influenza A (487)	23(1)
Acute myocardial infarction (410)	20(1)
Fracture Pelvis (808.8)	13(1)

Early Discharge (n Total=1,636)	n(%)
Aftercare following surgery (Circulatory system. Cardiac valve replacement, vascular bypass)) (V58.73)	433 (26)
COPD exacerbation (491.20, 491.21, 492.0, 492.8)	260(16)
Knee/hip joint replacement (V 43.65)	125(8)
Pneumonia (486)	122(7)
Acute Bronchitis/ Respiratory system disease NOS/ Other pneumonopathy (466, 519.9,516)	107(6)
Urinary tract infection (599)/ Acute pyelonephritis (590.1)	90(6)
Heart Failure/Complications of heart disease (428-429)	70(4)
Pulmonary embolism and infarction (415.1)	70(4)
Asthma exacerbation (491.03, 493.02)	57(3)
Bronchiectasis with acute exacerbation (494.1)	39(2)
Febril neutropenia (288.03)	22(1)
Limb cellulitis (682, 682.6)	16(1)
Fever and other physiologic disturbances of temperature regulation (780.6) (Cancer)	14(1)
Acute myocardial infarction (410)	11(1)
Influenza A (487)	11(1)
Surgical wound infection (998.5)	10(1)

Legend. Files correspond to diagnoses (within parenthesis ICD9-CM classification). Figures correspond to prevalence higher than 1%. Chronic Obstructive Pulmonary Disease (COPD). Discharges, total number of discharges (within parenthesis % total discharges, percentage of patients referred to the total number of discharges of Home Hospitalization and Early Discharge.

Table 2S. Risk factors of HH/ED readmissions at 30 days post discharge

	TOTAL			HH			ED		
Episodes, n	Readmissions n= 461	No readmission n= 3,704	p value	Readmissions n= 288	No readmission n= 2241	p value	Readmissions n= 173	No readmission n= 1463	p value
**Socio-Demographic** *(patient interview)*									
Age (yrs), m ± SD	73±12	71±15	<0,001¥	75±12	73±15	<0,001	72±12	68±15	0.001
Women, m ± SD	148(32)	1415(38)	0,011*	92(32)	919(41)	0.003	56(32)	496(34)	0.687
**Health Care resources** *(patient interview and clinical record)*									
Home care prior admision									
Primary Care, n(%)	52(11)	341(9)	0,133*	36(12)	242(11)	0.348	16(9)	99(7)	0.221
Palliative care, n(%)	11(2)	47(1)	0,050*	4(1)	34(2)	0.884	7(4)	13(1)	<0,001
Hospital, n(%)	7(1)	100(3)	0,137*	2(0,7)	78(3)	0.012	5(3)	22(1)	0.173
Without any suppot at home, n(%)	391(85)	3216(87)	0,170*	246(85)	1887(84)	0.676	145(84)	1329(91)	0.002
Hospital resources in previous 12 m									
Visits to ER, (Median, P_{25} - P_{75})	1(1-2)	0(0-1)	<0,001¥	1(0-2)	0(0-1)	<0,001	0(0-2)	0(0-1)	<0,001
Hospital admissions, (Median, P_{25} - P_{75})	1(1-2)	0(0-1)	<0,001¥	1(0-2)	0(0-1)	<0,001	1(0-2)	0(0-1)	<0,001
**Chronic conditions** *(clinical record)*									
> 1 chronic conditions , n(%)	378(82)	2806(76)	0.002	238(83)	1720(77)	0.024	140(81)	1086(74)	0.040
nº of comorbidities, m ± SD	4±2	3±2	0,006¥	4±2	3±2	0.411	4±2	3±2	0.001
Charlson Index, m ± SD	5±2	4±3	<0,001¥	6±2	5±3	0.001	5±2	4±3	0.003
**Main Diagnóstic Group, n(%)**									
Respiratory n(%)	151(33)	1323(36)	0,210*	100(35)	912(41)	0.042	51(30)	403(27)	0.618
Cardiology n(%)	86(19)	366(10)	<0,001*	69(24)	299(13)	<0,001	17(10)	67(5)	0.004
Post-surgery n(%)	63(14)	642(17)	0,048*	3(1)	17(1)	0.610	60(35)	625(43)	0.043
Oncology n(%)	62(13)	269(7)	<0,001*	49(17)	211(9)	<0,001	13(7)	58(4)	0.030
Acute illness n(%)	99(21)	1104(30)	<0,001*	67(23)	802(36)	<0,001	32(18)	310(21)	0.445
**Risk factors and treatments** *(patient iterview and clinic record)*									
Smoking (pack/yr) (Median, P_{25} - P_{75})	40(20-66)	36(14-60)	0,074¥	40(20-72)	35(12-60)	0.677	35(20-60)	37(15-60)	0.829
Active smoker, n(%)	33(7)	524(14)	<0,001	21(7)	282(13)	0.010	12(7)	242(16)	0.001
Ex-smoker, n(%)	249(54)	1649(44)	<0,001*	152(53)	999(45)	0.007	97(56)	650(44)	0.002
Sedentary lifestyle, n(%)	146(32)	892(24)	<0,001	102(35)	637(29)	0.013	125(72)	255(17)	0.008
Treatment prior admission									
Oxygen therapy at home, n(%)	65(14)	388(10)	0,104*	41(14)	259(12)	0.345	24(14)	129(9)	0.029
CPAP/BIPAP, n(%)	15(3)	156(4)	0,050*	10(3)	95(4)	0.242	5(3)	52(4)	0.529
nº pills / day, m ± SD	7±5	6±4	0,067¥	7±5	6±5	0.435	7±5	5±4	0.025
nº inhalations/day, m ± SD	0±1	2±3	0,002¥	2,6±3,9	2,1±3,3	0.053	1,8±4,2	1,5±3	0.017
**Patient's dependence factors** *(self-adnibistrated questionnaires)*									
Quality of life (SF-36)									
Physical status, m ± SD	30±13	34±13	0,007¥	29±13	33±13	0.905	31±12	35±12	0.913
Daily life activities (Barthel Index), (Median, P_{25} - P	100(80-100)	100(86-100)	0,060¥	95(80-100)	100(85-100)	0.996	100(85-100)	100(90-100)	0.001
Independent, n(%)	232(50)	2255(61)	<0,001*	136(47)	1291(58)	0.001	96(57)	964(66)	0.014
**Treatments during HH**									
Initiate nebulizer n(%)	124(27)	856(23)	0.030	89(31)	625(28)	0.180	35(20)	231(16)	0.138
Intravenous treatment n(%)	292(63)	1954(53)	<0,001	209(73)	1511(67)	0.078	83(48)	443(30)	<0,001
Required complex wound	84(18)	792(85)	0.131	27(9)	140(6)	0.040	57(33)	652(45)	0.004
Blood test	276(60)	1948(53)	0.007	182(63)	1339(60)	0.196	94(54)	610(42)	0.005
Blood gases	49(11)	480(13)	0.171	32(11)	352(16)	0.046	17(10)	128(9)	0.626
Forced spirometry	18(4)	400(11)	<0,001	15(5)	301(13)	<0,001	3(2)	99(7)	0.010
**Stays**									
**In-Hospital stay, days**									
Hospital stay (Median, P_{25}-P_{75})	1(0-2)	1(1-3)	0,001¥	0(0-1)	0(0-1)	0.732	4(2-7)	4(2-8)	<0,001
**Home stay, days**									
Home stay (Median, P25-P75)	6(5-8)	6(4-7)	0,006¥	6(5-8)	6(5-7)	0.119	6(5-8)	5(4-7)	0.004
**Total length of stay, days**									
In-hospital + Home (Median, P25-P75)	8(6-10)	7(6-10)	<0,001¥	7(6-8)	6(5-8)	0.240	10(8-15)	9(8-14)	<0,001
**Use of resources during HH/ED**									
Number of physician visits, m±SD	0,76±0.47	0,86±0,44	<0,001¥	0,76±0,48	0,87±0,45	<0,001	0,76±0,46	0,86±0,42	0.002
Number of nurse visits, m±SD	7±3	7±3	0,175¥	7±3	7±3	0.971	7,38±4,3	6±4	0.037
**Mortality**									
During 30 days post discharge, n(%)	36(8)	58(2)	<0,001*	24(8)	46(2)	<0,001	11(6)	10(0,7)	<0,001
**Continued care on HH discharge**									
Primary care n(%)	367(80)	3160(84)	0,001*	222(77)	1855(83)	0.010	145(84)	1305(89)	0.035
Palliative care n(%)	39(8)	187(5)	0,004*	24(8)	132(6)	0.100	15(9)	55(4)	0.004

Legend. Data are expressed as mean ± standard deviation for qualitative variables and number (percentage) for discrete variables. It is expressed as median (25-75th Percentile) in quantitative variables with extreme values. ¥Chi2 test or Fisher's exact

test were used for the comparison of proportions. *The Mann-Whitmey U test was used for variables not normally distributed. Home Hospitalization (HH), Early Discharge (ED), Emergency Room Department (ER), Body Mass Index (BMI), Continuous Positive Airway Pressure (CPAP), Bilevel positive airway pressure (BIPAP), The Short Form (36) Health Survey (SF-36).

Table 3S. Economic analysis at provider level

	2006	2007	2008	2009	2010	2011	2012	2013	2014	2015
n of discharges	303	347	441	444	385	394	444	432	422	559
**Cost**										
Administrative costs	14.900,00	14.900,00	14.900,00	14.900,00	14.900,00	14.900,00	18.625,00	18.625,00	18.625,00	18.625,00
Nurse costs	110.843,00	110.843,00	110.843,00	128.954,60	128.954,60	165.177,80	188.360,65	188.360,65	188.360,65	188.360,65
Physician costs	59.441,36	59.441,36	59.441,36	59.441,36	59.441,36	59.441,36	74.301,70	74.301,70	74.301,70	74.301,70
Drug costs	13.594,03	16.195,27	35.327,80	39.293,31	40.481,25	36.262,29	64.210,94	44.626,84	37.711,31	63.098,97
Consumables	711,77	1.298,76	1.736,28	2.619,59	4.204,01	1.477,61	4.938,88	2.744,10	2.480,01	3.602,14
Transport costs	10.722,43	12.061,68	13.658,64	19.589,42	15.818,67	11.757,27	8.450,46	9.182,64	9.856,60	10.676,50
**Total cost**	**210.212,59**	**214.740,07**	**235.907,08**	**264.798,28**	**263.799,89**	**289.016,33**	**358.887,63**	**337.840,93**	**331.335,27**	**358.664,96**
**Total reimbursement**	**295.225,02**	**340.802,58**	**439.200,72**	**438.105,90**	**384.033,65**	**372.846,14**	**397.450,20**	**379.167,60**	**379.167,60**	**392.817,60**
**Net cost of the program**	**85012,43**	**126062,51**	**203293,64**	**173307,62**	**120233,76**	**83829,81**	**38562,57**	**41326,67**	**47832,33**	**34152,64**

Table 4S. Analysis of other MAST dimensions

Domains	Results
**Patients' perspectives**	
Acceptance to participate	82% of the patients
Patients. Satisfaction<br>Caregivers. Satisfaction	99 % of the subjects reported that the treatment received was very good.<br><br>90% of the patients stated that they would repeat the experience if needed<br><br>94% of the caregivers stated that they would repeat the experience if needed
**Professionals' perspectives**	Initial common resistances to implementation from both Hospital and Primary Care staff markedly decreased over time.<br><br>Professionals of the HH/ED team showed high degree of satisfaction throughout the deployment period.
**Organizational and regulatory aspects**	The HH/ED enforced the bridging between hospital and community care throughout the study period and increased the transitional patient face after HH/ES discharge.
**Technologies**	Majors lessons learnt during the study period:<br>i) Interoperability at a health system level, across levels of care and among providers, is a must to optimize the program;<br>ii) Remote monitoring used by professionals visiting patient-home showed high efficacy and it was a source of cost-containment;<br>iii) Patient self-monitoring showed limited potential because of two main factors: the acute condition of the patient and the short available learning period, and,<br>iv) Interactive tools such as videoconference and the patient personal health folder seem to be useful to reinforce remote support, but we were unable to optimize these two ICT tools during the project lifetime.
